# How to dad? Italian fathers discuss fatherhood online

**DOI:** 10.3389/fsoc.2026.1692529

**Published:** 2026-04-17

**Authors:** Luca Falzea

**Affiliations:** Faculty of Political and Social Sciences, Scuola Normale Superiore, Pisa, Italy

**Keywords:** emotions, fatherhood, gender, masculinities, parenthood

## Abstract

**Introduction:**

This article examines how Italian fathers navigate masculinity, care, and parental wellbeing through an analysis of an online fathers' forum.

**Methods:**

The methods are qualitative: a critical discourse analysis of 180 posts and comments, that appeared on the forum between 2019 and 2025 was used to explore how participants negotiate their roles in a society that continues to privilege maternal caregiving, despite growing calls for gender equality.

**Results and discussion:**

The findings reveal a hybrid model of fatherhood that blends emotional engagement and caregiving with traditional elements of masculinity, such as protection and provision. Fathers in the forum share experiences of joy, anxiety, and loneliness, highlighting the emotional complexity of parenthood and the societal pressures shaping their identities. The forum provides a rare supportive space for men to discuss vulnerabilities and fatherhood outside the manosphere's misogynistic discourse, fostering community and emotional expression.

## 1 Introduction

In Italy discourse on fatherhood is rich and is often focused on how a country that still has a traditional mindset, especially when it comes to parenting (due to policy and cultural aspects), deals with a new emerging type of fatherhood. It seems that having a child in Italy still leads heterosexual couples through a path of (re)traditionalization (Naldini, 2016), as the policy landscape and the cultural and social expectations lead to a prominent role for the mother in childcare and for the father in paid work (Saraceno and Keck, 2011). Mothers are considered extremely important, especially in the first months of life of a newborn, due to their ability to lactate and for a general idea that a young infant has a special bond with the mother, while father's presence is not always considered necessary (Cannito et al., 2022). This political and cultural landscape favors a certain vision of what a heterosexual father should be and should do: being mainly a breadwinner for his family. Recently, as mothers and young women entered the labor force more massively and as discourse about gender equality has become more prominent in the country, fathers are also expected to be more caring and involved in their children's lives while the institutional landscape around them did not change to accommodate this expectation. Even if fathers nowadays in Italy seem to be more present in their children lives when compared to the previous generations, their role is often that of a playmate for their kids or of a helper for the mother, leaving the tasks of childcare to her, providing support when necessary (Fagiani and Ruspini, 2011). These characteristics of the Italian case could probably have an effect on parental wellbeing: if mothers are expected to be left almost alone in dealing with the children, they could be feeling isolated in their role; on the other hand, fathers are expected to increase their paid work commitment, cutting them off from the possibility to become competent as fathers and to build meaningful relationships with their children, meaning that they could lack important skills connected to care and nurturing and feel unprepared to be fathers. Starting from these considerations, the objective of this contribution is to look at an online space mainly for fathers where they can reflect on fatherhood and, through this analysis, observe how fatherhood is understood and discussed by fathers themselves in a space that is supposedly tailored to them. To do so I performed a Critical Discourse Analysis of an Italian forum for fathers, studying 180 posts and relative comments. Results show that changes in fatherhood practice and masculinity can coexist with more traditional and familistic approaches to childcare and that, in discussing fatherhood and the emotions connected to it, there is a space for the construction of a male gender-based community that is not united by hate for women. This community could be beneficial for fathers and favor parental wellbeing.

## 2 Fatherhood, emotions and masculinities

To introduce the data presented in paragraph 4, the following subparagraphs will thematize masculinities in relation to emotional expression. The possibility of hybridization of masculinity practice, through the incorporation of care activities in it, is particularly interesting to look as a key to interpret the discussions that happen within the forum examined. The first subparagraph will focus on what is masculinity and what does this process of hybridization entail; the second will present some perspectives on the sociology of emotions and its relation to gender and fatherhood; lastly the third will review the Italian context and the research done within it.

### 2.1 Hybridization of masculinities

According to Connell (2002), gender can be understood as a social practice: this definition makes clear the relationship between the pervasiveness of gender structures—as gender practice is constructed in historical processes—and the dynamicity of gendered projects, as people have always potentially the opportunity to revise their gender practice, that transforms with the passage of time (Connell, 1985). Practice of gender happens within a gender order—a system that allocates power according to the construction of gender (Connell, 2002)—and as such, masculinities and femininities can be understood only in their relational nature, as they are built on the relationship between different types of gender practice (Connell, 2018; Connell and Messerschmidt, 2005; Messerschmidt, 2019). These relationships are hierarchical, as modern capitalist societies are built on patriarchal dominance: to sustain the growth of capitalism someone has to perform the reproductive and care labor that sustains the productive labor of workers; capitalism, thus is based on a gendered division that relegates women to the reproductive labor and gives wealthy men the possibility to dominate other men as well as women (Federici, 2004; Bertone, 2024). This possibility of dominion can be understood as hegemony (Gramsci, 1971) or, as Connell and Messerschmidt (2005) put it, hegemonic masculinity. Many men will scoff at the notion of them being at the top of the hierarchical order in society: their perception of their privilege is lacking, most of them would feel they are being constantly at disadvantage or being bossed around by women in their life; this conceptualization of their position in society is both real, as they really perceive it, and false as men still hold most of the power in western societies (Kimmel, 2007). To comprehend this contradiction, it is useful to understand that hegemonic masculinity is not the one that is practiced by most of the male population, but it is rather a model of what a good man should be. Understanding masculinity and hegemony as a set of practices is thus useful to frame them as models of masculinity, as something desirable, but not necessarily attainable, by all men (Connell, 1990, 2018). Moreover, the focus on practices helps in understanding the dynamic nature of this hierarchy and the possibility of change within the hegemonic aspects of masculinity. Rather than imagining hegemonic masculinity as a set of attributes that define the hegemonic male, it should be understood as a relational concept, as something that gives power to men within a specific context and that is supported by other practices of masculinity and of femininity (Messerschmidt, 2019).

Connell (2018) observes how men can adapt their practice of masculinity whenever a fundamental aspect of hegemonic masculinity is not reachable: for example, college students who do not excel in sports, may try to become editor of their university newspaper, to retain status and dominance associated to hegemony without having to rely on their physical prowess. Considering masculinities as a practice means to be aware of the constant possibility of change in it. In the last decades women entered massively the workforce, as the ideal of “traditional” family with a sole (male) breadwinner and a stay-at-home wife was questioned. Men today in Western societies have changed to reflect the changes happening around them: their practice of masculinity in many instances has undergone a process of hybridization, as they incorporated in it elements that were traditionally considered as feminine (Bridges and Pascoe, 2013; Connor et al., 2021). This change could be read as a shift in masculinity practice that leads to the abandonment of the possibility of dominion allocated to men by the capitalist system. The concept of caring masculinities embodies this shift: caring males are those who completely rejects dominance to embrace care and engage actively in gender equality (Elliott, 2016). However, the idea of “caring masculinities” as an antidote to systems of dominion connected to hegemonic masculinity has been criticized as it seems that, in some cases, the adoption of care practices between men has broadened the boundaries of hegemonic masculinity, rather than substituting it (Hunter et al., 2017). In fact, this process of hybridization does not necessarily bring forward a change in gender relationships nor an abandonment of male patriarchal dominion: men can incorporate more “caring” or “feminine” traits and practices without challenging the gendered hierarchy; if anything the focus on defining such traits as “feminine” could reinforce this division and give them a status advantage in a society that apparently nowadays seem to value more these traits in men (Wojnicka and de Boise, 2025); contrary to what the concept of caring masculinities suggests, while distancing themselves from a traditional depiction of manhood, some men do not seem interested in challenging the system that still considers heterosexuality and practice of dominion as the best attributes for a man (Arxer, 2011; Bridges, 2014; Eisen and Yamashita, 2019).

### 2.2 Feeling rules, gender and changing fatherhood

The sociological interest for emotions can be traced back to the beginning of the discipline: Max Weber (1968) explanation of charismatic leadership is tied to a focus on the emotional aspects of being a leader; Marx (2004) description of workers' alienation and isolation can be read at an emotional level; in his study of the relationship between cities and countryside, Simmel (1995) speaks about how the different environments lead individuals to develop different types of emotional relationship; in his work on suicide Durkheim (1952) evaluates the emotional aspects connected to it; Martineau (1988), in describing how to observe ‘moral and manners' gives space to emotional aspects as well as moral ones connected to how one should conduct social research. More recently, in the last century, Hochschild (1979) developed a sociological approach to the study of emotions criticizing views that relegate the emotional level to the work of psychology and psycho analysis and connecting it instead to materialistic processes such as the division of labor and the social reproduction of society. Hochschild sought to understand how emotions are felt in connection to social expectations that exist in the form of feeling rules that, as the word suggests, guide the “proper,” expected emotional response to a vast array of different social situations: one person should feel joy during their wedding, sadness during a parent's funeral or rage in front of an injustice.

In common parlance, we often talk about our feelings or those of others as if rights and duties applied directly to them. For example, we often speak of “having the right” to feel angry at someone. Or we say we “should feel more grateful” to a benefactor. We chide ourselves that a friend's misfortune, a relative's death, “should have hit us harder,” or that another's good luck, or our own, should have inspired more joy. We know feeling rules, too, from how others react to what they infer from our emotive display. Another may say to us, “You shouldn't feel so guilty; it wasn't your fault,” or “You don't have a right to feel jealous, given our agreement.” Another may simply declare an opinion as to the fit of feeling to situation, or may cast a claim upon our managerial stance, presupposing this opinion (Hochschild, 1979, 564).

To Hochschild emotions are the results of a social construction that is mediated by not only ideology and convention but also by the capitalist system and the commercialization of feelings it brings (Hochschild, 2003): controlling both emotions and emotional labor (Hochschild, 1979) is a means to control workers' lives and alienate them further from others (Hochschild, 2003). Emotions are used to structure a gendered division of labor and of care work: guilt about not being a good enough mother or about child abandonment is used to limit women's possibilities in the workplace (Hochschild, 2012) and women and men within the workplace face different expectations around their emotional involvement or expression (Hochschild, 1983; Husso and Hirvonen, 2012). There are different ways to study the relationship between gender and emotions in sociology (see Lively, 2019 and Simon, 2014), but for this study Hochschild's perspective seems better suited as it keeps together structural aspects related to the division of labor and an interactionist stance that has its roots in Goffman (1959) studies of human interactions. In fact, looking at gender as social practice means that to understand the gendered experience of emotions and the expectations around it, the structural level is fundamental as much as the interactions that happen in what Connell (2002) defines as reproductive arena. Emotions and their expression are connected to the gender projects of men and women and, in turn, affect also the attitudes toward and practice of fatherhood and motherhood. Women are expected to be nurturing toward their children and to feel guilt when they can't be around them (Hochschild, 2012) and on the other hand, the possibility to express a larger and more “feminine” set of emotions and to perform acts of care is often considered a sign of the hybridization of masculinity (De Boise and Hearn, 2017; Elliott, 2016).

The relationship between masculinity and emotions, in fact, has often been studied with a focus on how patriarchal structures hinders the possibility of emotional expression for men leading to mental health issues (Monaghan and Robertson, 2012a,b; Berke et al., 2018; Prakash Tode and Shukla, 2023; Guglielmelli, 2025) and higher suicide rate than women (Cleary, 2012; River and Flood, 2021). Due to patriarchal expectations around their gender, it seems men have a harder time in asking for psychological support and in recognizing their psychological needs (Günaydin, 2024). This may lead to adversities for heterosexual men in their romantic relationships, whenever they tend to ignore the emotional investment in their coupled lives, leaving the burden of the emotional labor to their partners (Duncombe and Marsden, 1995). Other research focused on the way emotions are displayed within male-only spaces and how this display is connected to the practice of masculinity (Oransky and Mareck, 2009; Underwood and Olson, 2018). However, critical approaches noted how men's possibilities in expressing emotions, rather than being negated or limited seems to simply be different from women's (De Boise and Hearn, 2017): some of these studies unveil the connection between emotional expression and gender based violence, noticing how men are perfectly capable of feeling aggression, dread and anger (Tereškinas, 2023), justifying their violence against women using feelings of nostalgia for a “disappearing male-dominated world” (Botto and Falzea, 2025) or instrumentally using shame and guilt to explain violent behavior (Gottzén, 2016). In summary, men's emotional expression is limited by socialization to hegemonic masculinity (Tereškinas, 2023) and (re)discovering the possibility of emotional vulnerability (Holmes, 2015; Botto and Gottzén, 2023) and of performing care (Elliott, 2016) could help men to emancipate from a harmful relationship with their emotions and practice of masculinity.

Transition to fatherhood could be one of the catalysts that guides this process of hybridization of masculinity. Hybridization is used in this context to underline how this process is not linear: “modern fathers” are more involved in their children's lives, however, this does not necessarily revolutionize how men and women care for children in heterosexual couples. In the last decades fatherhood has changed and become more varied (Hochschild, 1994; Andreasson and Johansson, 2016), more individualized and less traditional (Williams, 2008), even though a change in attitudes toward fatherhood in heterosexual couples does not always translate in a more equal division of care work with the partner, as the mother remains the principal caregiver (Wall and Arnold, 2007; Cannito et al., 2022). Even though fatherhood in Western countries still leads to a bigger investment in paid work for men (Cannito, 2022), especially in dual income couples, fathers often deploy strategies to balance both care and work life (Gottzén, 2011; Bataille and Hyland, 2022). However, it must be stressed that the organization of childcare within families is extremely context dependent: it is not only a matter of cultural attitudes toward motherhood and fatherhood but also a matter of how the labor market is organized—is there the possibility for fathers to take paid leave for caring for their children?—and the general recognition of the fathers' role within society (Saraceno and Keck, 2011). For example Kosakowska-Berezecka et al. (2022) analysis of data from 62 countries shows that gendered self-views are dependent on the level of egalitarianism of the country; also, while both possibility of paternity leave and attitude toward gender in men seem to be related to the involvement of fathers in their children's lives, the second one seem to be more prominent (Olsson et al., 2023). What seems to be happening in many western societies is a fundamental shift in the expectations around fatherhood and a consequent change in attitudes toward it, with intimacy being given a more prominent role in father-children relationship and the consequent abandonment of a rigid breadwinner/provider model (Dermott, 2003, 2008). However, facing a context that is not always welcoming of different expression of masculinities and fatherhood, men often adopt both their own fathers' more detached style of parenting and a more nurturing one, adding what they perceive as manly elements to their practice of care (Miller, 2011; Hauser, 2015; Naldini, 2016; Randles, 2018; Mercuri, 2021).

### 2.3 The Italian context: gender, masculinities and the family

According to the Italian National Institute for Statistical Research (ISTAT), in 2023, 52% of working-age women in Italy were working, compared to 70% of men in the same range (ISTAT, 2023a). ISTAT also certified that gender stereotypes are still largely present in Italian society, as more than a third of male respondent believes that women can refuse non-consensual sex if they wish to, 20% believes that it is more important for men than women to have a fulfilling career and 24% believes that women can be fully satisfied in life only through the experience of maternity (ISTAT, 2023b). Italian women are still the main caregivers of young children (Saraceno and Keck, 2011; Solera, 2012; Naldini, 2016; Carriero and Todesco, 2016; Cannito and Scavarda, 2020) and of elderly parents, often at the same time (Saraceno, 2003; Naldini, 2014; Torrioni, 2015). This data tells of a country that, differently from other European similar countries, still struggles to change its attitudes toward gender, especially when it comes to maternity (Bertolini et al., 2016). Cannito et al. (2022) research on Italian families with children of different genders shows that families attitudes toward gender equality changed compared to the previous generation: parents try to steer away from their families of origin's views on gender, and, like their children, they believe in equal opportunities for men and women. However, practice of gender is still somewhat traditional as care work—and the organization of it—is mostly demanded to women. Italy, compared to other European countries, still seems to struggle in changing gender practices and attitudes toward gender equality (*ibidem*) and the transition to parenthood still seems to lead to (re)traditionalization of the couple, with men investing more time in paid work and women in care work after having their first child (Naldini, 2016).

When it comes to fatherhood in Italy, the context described above of expectations around gender and maternity is to be taken in account: Italian fathers deal with a society that still largely expects child care to be carried out by women and that not always recognize their role as fathers beyond traditional expectations connected to it; on the other hand, Italians' view on gender relations is, albeit slowly, changing (Carreri, 2014; Bainotti and Torrioni, 2017; Cannito et al., 2022), meaning that Italian fathers are living within a contradictory movement as they are both expected to be more involved but there are not a system and a context that support this greater involvement. In Italy, paid and mandatory paternity leave amounts to 10 days, compared to 5 months of mandatory and paid maternity leave. Regardless, there are obstacles that impede the fruition of this right for fathers: while pregnancy make the necessity for a maternity leave visible, fathers must self-report the birth of their child and subsequently request for leave; often, employers, especially in the private sector, ostracize the request, as there is still an expectation that women would do most of the care and, as such, paternity leave is deemed unnecessary; due to this expectation, many fathers themselves prefer to invest more time in paid work, instead of requesting paternity leave: in this sense, their practice of fatherhood still entails a provider or breadwinner model (Bosoni and Mazzucchelli, 2018; Cannito, 2020). While Italian fathers' identification with this breadwinner model is still strong (Bosoni, 2014), in the last decades they have changed their perception of their role to incorporate in it elements of care and intimacy, as younger generations of fathers abandon more traditional practice of fatherhood (Ruspini, 2011; Fagiani and Ruspini, 2011; Bertone et al., 2016; Maridaki-Kassotaki et al., 2017; Bertolini et al., 2019; Mercuri, 2021; Marchesini and de Rose, 2025). This change in fatherhood practice, just like the ones in masculinity practice, does not necessarily linearly guide men from a traditional stance toward gender to an equality mindset, but rather seems to give life to a hybrid model of fatherhood where aspects of emotional involvement coexist with more traditional elements of the breadwinner father and of a traditional view on gender and gender relationships (Magaraggia, 2012; Musumeci et al., 2015; Bertone et al., 2016; Cannito, 2020; Mercuri, 2021). This hybridization seems to favor more certain aspects of parenting, such as playing and being helpers for the mom, that remains the main caregiver (Fagiani and Ruspini, 2011; Della Puppa and Miele, 2015): this role of fathers as playtime companions is also present in certain depictions of fatherhood (Cannito and Mercuri, 2021a). Lastly, another interesting aspect of this contradictory landscape regards what happens in case of separation or divorce: fathers' rights, in some instances, are instrumentally used to attack women freedoms and feminist policies (Cannito and Mercuri, 2021b).

## 3 Methodology and ethical issues

The objective of this work is to look at an online space mainly for fathers where they can reflect on fatherhood and through this analysis observe how fatherhood is understood and discussed by fathers themselves in a space that is supposedly tailored to them. To do so I analyzed a forum for Italian fathers as case study: the architecture of the platform used by these fathers stimulates a discussion around specific topics, in this case fatherhood, and is horizontal enough to have the most important themes for the users emerge with little moderation. It seems that fathers often struggle to find spaces specifically devoted to fatherhood offline, that's why a virtual forum with the guarantee of anonymity is an interesting and easy to access space to observe the relationship between fathers, their worries and their feelings around their children; moreover, social media dynamics and their affordances or the diffusion of the content can influence the way discourse around fatherhood is constructed as shown by social psychology research on social media platforms (Puryear et al., 2024). Through a critical discourse analysis (CDA; Fairclough, 2013; Forchtner, 2021) of the sample the contribution aims at answering the following research questions: what kind of narration of fatherhood emerges from the discussions between fathers within the forum? What emotions are connected to this narration? How is this discursive practice of fatherhood connected to practice of gender?

The sample consists of 180 posts and related comments that appeared on the forum from 2019 to 2025; this sample (180 posts) comprise all the posts that appeared in the timeframe examined, minus the ones (around 20) that had received no comments, that were excluded as the objective was to analyze discussion around fatherhood. The forum has been more active during 2019 and the first months of 2020 and in fact most of the posts analyzed (90 posts) are from that timeframe; in more recent years the forum has progressively been abandoned, even though it must be noted that participation in it was never a massive phenomenon, with the average answer rate to the posts being around 10 comments. The discussion seems to have been reduced during the COVID-19 pandemic and resumed, with less intensity, after the pandemic in 2021 and then declined again after 2022. I used coding to develop a framework to look at the way fatherhood and masculinity is built through the discourse between participants to the forum: the first round of coding was aimed at describing the themes that emerged from the discussion posted on the forum: it seems that a large majority of the posts are either asking for help or discussing the difficulties of dealing with newborns and the emotions related to fatherhood, while a minority of them are related to suggestions about good childcare equipment. Thanks to this first round of coding, I tried to understand how different categories of fatherhood emerge in the narration of fathers and how they are co-constructed through the interaction between them; these categories are already recognized in research on Italian fathers: the involved father, the playmate and mom's helper (Della Puppa and Miele, 2015; Cannito, 2020; Cannito and Mercuri, 2021a). The other element that I analyzed was the emotional aspects of the posts: stress, anxiety, tiredness but also joy, wonder and happiness were shared through the analyzed posts. The use of codes is intended to categorize the discursive practice observed in the forum. With CDA and the focus on discursive practice the aim of the article is to understand how discourse in the forum relates to gender structures and expectations around fatherhood in Italy and how the interaction between participants leads to negotiating the cultural meaning of what is supposed to mean being a father.

The data presented were collected from a public forum: the author decided, in order to guarantee the maximum level of privacy for the commenters, to store the data in a way that it would make impossible to know the username of the commenter even for him. To further protect the privacy of the participants, as the forum is publicly accessible, some personal details that would make the authors of the posts recognizable were not included and—even if the quotes written in the article are not the original as they have been translated from Italian—the comments that were included in the article were slightly changed (in a way that would not change the original meaning) to avoid the possibility of them being pasted in a browser search bar to find the source material. As a final protective measure, the name of the platform itself was not included as it is not relevant for the analysis. For these reasons, the demographic information about the participants was inferred by their comments, as the author did not observe the users' public profiles. It appears that most of the participants are males, between 30 and 45 years old, with young children and in heterosexual relationships.

## 4 Becoming a dad, discussing fatherhood

In the following subparagraphs a selection of posts from the forum is presented and analyzed. The conversation in the forum was entirely in Italian and for this reason all the posts included in the article have been translated by the author.

### 4.1 How to dad? Facing changes

The transition to fatherhood is narrated by the participants to the forum as a monumental event that shocks their lives, their priorities and their relationships with their partners and friends. Answering a post about the birth of the first child, this father describes the changes one experiences when becoming a parent.

Is not only about their first months of life: a child brings new elements that make your routines completely different from before. You will start to notice the difference between your friends with children (more similar to you in the way they spend their days) and your childless friends (you won't meet them often, even if you care for them). It's inevitable and to me it's about “becoming an adult.”

Parents are “naturally” more comfortable with other parents, as they can understand their struggles and the needs that arise when raising a baby. More interestingly is the connection traced between transition to parenthood, that apparently entails renouncing a certain type of friendships, and the transition to adulthood.

We are the only ones between our friends with a baby. We see the younger ones seldomly, as they stopped inviting us to their outings, while the older ones are okay to have stay-at-home nights together.

Literature on transition to adulthood in Italy underlines how the process has become increasingly less linear and that parenthood is still an important element in defining the transition and in perceiving oneself as an adult (Santoro, 2020; Gambardella et al., 2021). In this connection between abandoning “childish” friendships and restructuring routines to accommodate the newborn there is an undertone of traditional values: being an adult means to take part in the reproduction of society and to dedicate oneself more to family than friends.

Making space for a newborn entail both material activities—such as preparing the bedroom and making it safe for the child or deciding what type of car seat to buy—and psychological ones, such as restructuring ones' expectations and routines, as narrated by this father.

At the beginning everything is crazy: there are no calendars, you need to organize every little thing, even a walk, that should happen between feedings. It's hard and we don't always make it, but I don't feel it is problematic. Before becoming a dad, I used to participate in this forum to talk about paternity leave. I think I am truly privileged in working for an employer that allows a generous leave even for fathers. This helped me relax, to think about my child without worrying about work. […] It's hard at the beginning because the baby won't sleep and you must be prepared to wake up during the night.

In this recount the experience of parenthood is presented as a life altering one and there is an interesting element of discussion about balancing fatherhood with work life: the words of the post signal a shift in what being a father means as it calls for a paused work life, instead of a renovated investment in it, even though support of the mother as primary caregiver is still present (cfr. 4.2). This shift is made possible by a context that supports and welcomes involved fatherhood, that the participants in the forum recognize as rare and privileged.

Not everyone, however, believes that the restructuring following the birth of a child to be this severe. In some cases, the participants advocate for the necessity of balance in welcoming changes but also keeping elements of their lives pre-birth.

My wife and I have school aged children, and we are in our late thirties. We have a life beyond parenting and family. We like to meet friends, we organize so one of us can be at home, if the other wants to go out. We are gym rats, and we organize our time in order to go to the gym thrice a week. We try to organize our house to accommodate our hobbies, and we find ways to spend some time together alone.

This attention to one's personal needs and space is considered necessary to be well-adjusted parents and to provide a safe environment for the children. Renouncing too many hobbies or interests would generate frustrated parents that dump these negative feelings onto their children.

It is important to have some me-time, no matter what. If you adopt the mindset of “I am a parent, I can't have fun” then you will start despising the world around you.

We share care duties: we like trekking and going into nature and we try to bring our child with us when we do these activities. She is older so it's easier to bring her along. We organize in a way that if one of us wants to do something the other helps. In order to avoid frustration as a parent you will need to take care of your needs and hobbies.

The participants to the forum recognize that women are more often than men pressured about their dedication to family and children and discuss of strategies to resist such pressures and to push their partners to adopt a balanced relationship between childcare, work and leisure time.

External pressure, rather than biological reasons, push women to feel like they need to dedicate themselves only to the family: dads should make their partners take more time for themselves. It is better to have a happy partner, that is important too.

The “best for the baby” (Bertolini et al., 2016) remains the guiding principle behind the fathers' actions and decisions, but the prominence of the mother is challenged without necessarily being substituted by a more nuanced approach to parenthood as a whole: parents and the nuclear family is still considered the best place for children to be in, the parents still need to accommodate to give their children the best chance in life without too much external help, but the greater involvement of fathers allows for the easing of the mothers' burden.

### 4.2 Still mom's helpers or playmates? Signs of involvement

The participants in the forum do not want to replicate a traditional model of fatherhood and they do not want to renounce intimacy with their children and the practice of caring for them. When discussing the possibility of being a stay-at-home dad, the participants talk about the reactions from their workplace to this decision when they took it and their feelings on the “stigma” connected to it.

I was home from work with my children in two instances because I did not want to leave them with my parents. In the first case, I worked jobs that were not good, while in the second I had to renounce some work opportunities. I was working from home while taking care of the children and my wife had to take more domestic responsibilities for this. I think being home with your children, if you can afford it, is the best: you just need to handle the stress and it's perfect. Your children won't be with their grandparents all the time nor would be—even worse—sent to be with strangers, no matter how good and qualified. I don't care about what people think, I want to stay with my kids.

In this description of involved fatherhood that rejects the idea of the breadwinner model, there is still a traditional undertone signified by the familistic approach: family is the ideal place for a child to grow and spending time with the parents is ideal compared to daycares or preschools; this follows the traditional idea of the prominent role of the mother as the best outcome for the child (Bertolini et al., 2016), only including fathers as possible primary caregivers.

If you want to stay at home, you either need to have a real “smart” job—assuming you are still working—one where you work by deadlines and objectives, but in Italy this doesn't happen often, even though we had a chance to make it happen with COVID. Otherwise, you need to have some ways to earn money from passive investments or have a wife that has a good paying job. It is hard but spending time with your children is special: I would quit my job to stay with my kid if I had the means to do that.

In rejecting the absolute necessity to be a provider, these men refuse one of the main characteristics of traditional fatherhood and this signals a shift in their practice of masculinity and of fatherhood. These fathers hypothetically would have no issues in having their partners supporting the family financially, while they care for the children and this is an interesting subversion of expectations of roles within heterosexual couples: while women started working in salaried job, men did not as massively start taking care of household chores and care work (Esping-Andersen, 2009; Bertolini et al., 2013). If hypothetically these fathers speak of a very involved fatherhood, the actual practice of fatherhood they perform often has slightly different contents. For example, in the first weeks of life of the baby, the father's role, in the words of the participants, is mainly that of taking care of practical needs of the mother, who is recognized as the principal caregiver, especially due to breastfeeding.

Breastfeeding is pain and hardship and can lead to a depressed mother, so you as a father should support her and cater to her needs. Mother's milk is fundamental for the baby: of course if there is no possibility to breastfeed, formula is fine, but the best for the child's immune system is breastfeeding.

I supported my wife's decision to stop breastfeeding because I thought is more important to have a mother that is well-adjusted and not depressed around the baby. The pressure to breastfeed, especially by other women, is too extreme sometimes.

Fathers must be strong in supporting mothers that are going through the biggest changes in their bodies to provide the child with the best outcome possible (breast milk vs. formula). In this sense, fatherhood seems to be relegated once again to the simple act of help and support of mothers, when the child is very young. However, this support seems to be different from that of a passive helper who simply follows the mother's lead: taking care of the needs of the partner is something that is considered extremely important in general, as it is a way to protect the baby.

You should take into account your wife's needs: if she complains about caring for the baby, maybe she is asking you to step up. Sometimes as parents—both mothers and fathers, I experienced it—we feel that we always need to be present for being a good parent, but if we feel negative emotions like stress we risk transmitting them to our kid. For this reason, you need to make sure your wife is okay. I know it doesn't seem much, but I know of fathers that refuse to do simple acts of care because “they already did it yesterday” or because “they need to work.” Those are terrible humans.

The support—at least when the baby starts to grow and is perceived as less mother dependent—is mutual as partners should be attentive of one another to be sure they can provide a stable environment for the child. In different occasions the participants to the forum explain how important it is to cultivate the relationship with the partner and to favor an open communication to avoid creating a strain in the parental couple. It is, once again, interesting that the words used seem to refuse a breadwinner model: bad fathers avoid caring for the baby to dedicate more time to paid work.

Lastly, the participants in the forum do not seem to be interested in simply being playmates for their children, even though playtime is at the center of many of their discussions within the forum. Playtime is, however, considered an act of ordinary care, rather than the father's main responsibility and a way to get to know the children better and share some of one's passions and hobbies with them, as shown by a discussion some fathers had on video games and how to play with young children.

### 4.3 Emotions and loneliness

All the fathers express joy and excitement about their fatherhood experience, often defining fatherhood as the best and most fulfilling experience of their lives. This again marks an interesting shift from a model that puts breadwinning as the prominent feature of fatherhood and salaried work as the most important arena of success for the construction of masculinity.

Fatherhood is the most special thing that ever occurred to me. My life changed and accepting it made me better and now I want to work hard to give my son a future.

There is this little creature that is completely dependent on us, my wife and I. I can't believe I am a dad, it feels like a dream. I used to go visiting my friends and family with children, left after a few hours, got back at my own place without the kid. Now I have a kid of my own, at my place and I won't leave them ever!

While the first comment has elements in line with a breadwinner model (work here is intended as salaried work), it still characterizes fatherhood as a fundamental event; in the second salaried work is not mentioned and the author underlines the joy connected to parenthood and the excitement deriving from having a child of his own, that he never wants to leave. Fathers participating in the forum do not think that their experience is devoid of challenges, but they overwhelmingly agree that the positive sides outweigh the negative ones.

At the beginning it was terrifying, and I was sure we wouldn't have any other children, but now our child is older, and we want to have another one. It is hard, but it is so for a small period of time, and you soon forget about the negatives.

The negative emotions associated with the transition to fatherhood are mainly anxiety and fear of not being able to provide or ensure the safety of their children. The following request for help summarizes how many of the participants feel when it comes to anxiety.

I am used to overthinking and I am a little anxious, so I need all the information on a situation to understand how to proceed in it. Since I had my child, I have been more anxious and my wife doesn't always make it better, as she makes me even more anxious. How do you deal with this and try to stay patient?

Many answered the previous commenter questions underlining the importance of communication with the partner and also sympathizing with his situations as they believe that society around them does not accommodate these feelings of inadequacy in fathers, as they are often ignored. In fact, struggles also come from the relationship with the context and with the friends. Many complain about feeling lonely and isolated, especially from childless friends, as their children and taking care of their wives are now the epicenter of their universe.

Q: I live for my daughter now, I think it is natural. I help my wife and I want to make her burden lighter. I feel like I am distant from my friends, did you guys feel similar in the first months? A: Your firstborn comes suddenly into your life. It's normal to feel pressured and like you don't have personal space or control of your time. Or maybe like you have no time at all. It is just a feeling though, even if anyone reacts differently to it.

These feelings of detachment from oneself are often presented as a form of depression. Many fathers in the forum suffered from it and seem to believe that the root of it is an unwelcoming society that alienates them from their children.

Fatherhood is being considered, finally, as an important figure for the growth of the children, we are not expected to simply go to work, but we are expected to care for them from day one. Once they described us as women if we took care of our child, but I have never cared for such nicknames. I wanted to be active part of my child's life. Even if things changed within the family, in Italy society doesn't really change. We just had a small bump in paternity leaves day, nothing to be excited about. It seems that most people around us think that “men need to be masculine” or macho and I find this annoying. This context gives me tiredness, stress, frustration, all feelings that are not connected to how my family is but how the society I live in is.

One interesting aspect here is how the family is presented in contraposition to society: similarly to what Cannito et al. (2022) notice, family is often presented as something that is above reproach in a society that instead is problematic when it comes to gender relations and expectations around gender roles. Apparently, families and individuals move at a faster pace than society who pressures men into roles they do not recognize anymore, such as that of the breadwinner father.

Around me, in society I feel like people do not really see fatherhood for what it is today. How many times I heard “act like a man,” but I feel like my child is both mine and my wife's, we are both responsible for them.

For men that are involved in care, this pressure could often translate in a depressive state, according to a commenter who shares his experience.

I don't care what others think. However, I still felt strongly depressed after my little ones were born. After the first, my job was so demanding that I didn't have time to be there for my child like I wanted, up until their third birthday.

These feelings often come from a difficulty to bond with the children: these fathers often want to be involved but they, especially in the first weeks of life of the children, don't have many chances to create a strong bond, according to them. In expressing this difficulty, the participants in the forum show one of the rare signs of gender essentialism, as they connect this difficulty to the different biological roles mothers and fathers allegedly have.

It was hard to get used to my life as a father, as a newborn brings so many changes and we, as men, don't have the hormonal and physical properties that guide us in this change naturally like moms do. […] In my experience I sometimes felt like an extra: mom breastfeed and what do I do while she does? […] We, in today society, are used to have more leisure time and a kid challenges this in a way that no other experience can: it's not hard to have a newborn, you don't need to do anything special, they just need you there. It's simple but not easy.

In this instance it's particularly interesting how the reification of gender differences influences the perception of fathers: masculinity is built around the possibility of acting, of doing something “concrete” (Connell, 2018) and fathers seem to struggle when the baby is too little to engage in any activity. This reification is also used to justify feelings of inadequacy, as women “are simply better at it due to their nature.” When the child grows up, these feelings of depression and detachment can be resolved through a major involvement in fatherhood.

My child and I started bonding when he was almost four. I changed my job, because I wanted more time outside of it and less stress. From then on, my child started to be more around me, and I had more time for him, which was great. We started connecting from there, I hope it will last forever. I became his role model, even now that he is a preteen and he is building a life beyond me he is still close to me.

Fatherhood seems to be able to be actually involved when the children can participate in activities that require movement and when the society around fathers allow them to practice a more involved version of it, allowing them to take time from work to be with their families.

## 5 Conclusions: new fathers, emotions and parental wellbeing

The data collected for this article present a very small case study that is in no form representative of Italian society: the fathers participating to this forum do so with the desire to speak about their involvement as fathers and they are thus an extremely self-selected sample, were more traditional understanding of fatherhood are absent. As such the analysis provided cannot suggest trends in the changing of fatherhood or parenting in Italy but is certainly useful to unveil dynamics in the way fatherhood becomes involved or hybrid. With regards to the type of discourse around fatherhood that emerges from the forum, the men participating in it show that fatherhood can be “new” or involved while also retaining some traditional elements, as shown already in other research on the argument in the Italian context (Magaraggia, 2012; Naldini, 2016; Cannito, 2020, 2022; Mercuri, 2021; Cannito et al., 2022). The online space observed was interesting as it was mainly inhabited by men, fathers, that had the chance to interact about their experience on their own. The discussion was mainly centered around children and care, and their feelings and the general tone was always respectful and supportive. This way of interacting is in line with the narrative of fatherhood the participants support: fatherhood to them is mainly an act of care and they are actively trying to emancipate themselves from a model of breadwinner father, detached from his emotions.

When it comes to emotions connected to fatherhood, it can be noted that they play an important role in defining this type of fatherhood: the participants share their fear, anxiety, joy about being fathers and their disdain for a society that, by limiting their emotional expression, limits also their possibility of being an involved father, often relegating them in time-consuming and stressful jobs. Interestingly, while this progressive stance on the meaning of fatherhood is accompanied by a critique to gender roles and stereotypes, it also coexists with more traditional ideas of family and adulthood. The solution to a model that puts too much burden on the mother is to share that burden equally within the nuclear family: both parents are required to dedicate themselves completely to their children as the family is the only place where a child can grow well. While there is a consensus on the fact that it is important to take care of oneself mental health and leisure time, especially within the couple, these efforts are always made with the best interest for the children in mind. Following this reason, the participants often trace a distinction between a society that is still patriarchal and unjust and the family that instead is more equal on the grounds of gender. In this sense, while promoting a new idea of fatherhood they still support a typical Italian model of familist care (Saraceno and Keck, 2011).

When it comes to gender, fatherhood seems to open the participants to the possibility of revising their practice of masculinity to incorporate in it acts of care (Elliott, 2016). Together with these new elements in their practice of gender, it still seems to persist an idea of man as someone who needs to protect and support partner and children, especially during the first stages of life of the babies. While fatherhood gives these men the possibility to explore care, they still reify gender differences when it comes to the way this care is performed. When the children are very young, they act more as support for the mother, and they become more involved as they grow up and can share more activities with their fathers. Thus, in the narrative observed within the forum the involved father model is prevalent in a hybrid version that still retains elements of the “mom's helper” model, breadwinner model and playmate model. As noted, most of the posts analyzed were shared before the COVID-19 pandemic and discussion in the forum resumed in the aftermath of it. Italy was one of the first European countries to be largely affected by the pandemic and imposed restrictions on the freedom of movement for its citizens. In a familistic country such as Italy, the lockdown mandate exacerbated the expectation that parents (more often mothers) were to take care of their children with little to no public policy support, with effects on parents' wellbeing and mental health (Cannito et al., 2021). The pandemic partially restructured fathers' involvement and care practices as shown by comparative studies (Petts et al., 2023). In Italy this newly involvement for fathers, while having positive effects on children's wellbeing (Mangiavacchi et al., 2021), did not restructured the normative gendered divisions of care work (Cannito and Scavarda, 2020). Moreover, the absence of public policy that would sustain fatherhood involvement (e.g., paternal leave) risks making this major involvement seen during the pandemic an extraordinary event, rather than a new normality (Margaria, 2021). Fathers in the forum rarely discussed the pandemic and its restrictions directly, but given the distribution of the posts timewise, it could be possible that the pandemic restrictions gave them the possibility to be more involved as fathers and thus gave them the push to reflect more clearly on their involvement as parents and to express more freely their desires about it.

The possibility of being involved fathers and of avoiding stigma is presented by discourse within the forum as beneficial to parents, as it is an antidote to depression, fear and anxiety connected to parenthood. Spaces like this, tailored to fathers, could help in sharing this kind of experience with negative emotions connected to fatherhood and thus create a sense of community and belonging. It's interesting to note that what the men participating to this forum did was creating a community that while primarily based on parenthood, was also particularly focused on their experience of gender and the (rare) disadvantages that comes from being a man, such as less access to parental leave and a general assumption about a minor involvement in caring activities as fathers and, as such, an expectation to increase or to not change their involvement in paid work after having a child, despite their desire to share more time with their children. As such this community is an example of the possibility for men to construct a sense of belonging to their gender, recognizing the faults in a patriarchal system that hinders their emotional expression and their possibility of performing care, without tearing down feminism and women in the process, like often happens within the manosphere, where men vulnerabilities are exploited to foster misogyny (Botto and Gottzén, 2023; Cannito and Mercuri, 2021b; Perin and Ferrero Camoletto, 2025). Possible reasons of this tendency in the community could be related to the main themes of the forum, that privilege a narration of fatherhood focused on the relationship with the children, rather than with significant others, other women or society in general; also, the ruleset provided in the community's description highlights the necessity to use civil language and avoid vulgar modes of expression and could indicate an attention on self-restraint and moderation that could also have effects on the kind of public the community attracts. Future research could possibly explore more deeply the elements that make certain communities, such as this one, less violently misogynistic and their potential in developing a sense of belonging to the male gender that is not necessarily contrary to feminism and women.

## Data Availability

The raw data supporting the conclusions of this article will be made available by the authors, without undue reservation.
